# Unraveling Acute Cardiorenal Syndrome: Predictors and Consequences in Acute Heart Failure

**DOI:** 10.3390/jcm14072270

**Published:** 2025-03-26

**Authors:** Georgios Aletras, Maria Bachlitzanaki, Maria Stratinaki, Emmanuel Lamprogiannakis, Stylianos Panagoutsos, Konstantia Kantartzi, Theodora Georgopoulou, Ioannis Petrakis, Emmanuel Foukarakis, Yannis Pantazis, Michael Hamilos, Kostas Stylianou

**Affiliations:** 1 Department of Cardiology, Venizelio General Hospital of Heraklion, 71409 Heraklion, Greece; maria.stratinaki@gmail.com (M.S.); mlamprog@yahoo.gr (E.L.); theodgeo7@gmail.com (T.G.); mfouk@hotmail.com (E.F.); 2 School of Medicine, University of Crete, 70013 Heraklion, Greece; mchamilos@uoc.gr (M.H.); kstylianu@gmail.com (K.S.); 3 Second Department of Internal Medicine, Venizelio General Hospital of Heraklion, 71409 Heraklion, Greece; mariabachlitzanaki@gmail.com; 4 Department of Nephrology, University General Hospital of Alexandroupolis, 68150 Alexandroupolis, Greece; spanagou@med.duth.gr (S.P.); kkantart@med.duth.gr (K.K.); 5 Department of Nephrology, University General Hospital of Heraklion, 71500 Heraklion, Greece; petrakgia@gmail.com; 6 Institution of Applied and Computational Mathematics, Foundation of Research and Technology-Hellas, 70013 Heraklion, Greece; pantazis@iacm.forth.gr; 7 Department of Cardiology, University General Hospital of Heraklion, 71500 Heraklion, Greece

**Keywords:** acute heart failure, acute cardiorenal syndrome, acute kidney injury, renal outcomes, mortality, chronic kidney disease

## Abstract

**Introduction:** Acute cardiorenal syndrome (ACRS) is a common complication of acute heart failure (AHF), leading to worse outcomes and therapeutic challenges. This study aimed to identify clinical parameters associated with ACRS and evaluate its impact on prognosis in hospitalized AHF patients. **Methods:** This prospective observational study included patients hospitalized for AHF at the Venizelio Cardiology Department from February to November 2023. Demographic characteristics, comorbidities, medications, laboratory and echocardiographic parameters, hospital stay, and in-hospital mortality were recorded. Patients with incomplete data or end-stage chronic kidney disease (CKD) were excluded. Survivors were followed for six months to assess renal function changes, readmissions, initiation of renal replacement therapy (RRT), and mortality. ACRS was defined as a serum creatinine increase of ≥0.3 mg/dL or ≥1.5 times baseline. **Results:** Among 218 hospitalized AHF patients, 112 (51.3%) developed ACRS. These patients were older, had higher CKD prevalence, worse New York Heart Association (NYHA) functional class, lower hemoglobin, and higher N-terminal Pro-B-type Natriuretic peptide (NT-proBNP) levels. Multivariate analysis identified CKD stage (OR 2.30, 95% CI 1.64–3.23, *p* < 0.001) and creatinine change on admission (OR 3.53, 95% CI 2.02–6.18, *p* < 0.001) as independent predictors of ACRS. ACRS was associated with higher in-hospital mortality, longer hospital stays, increased vasoactive medication use, worsening renal function, and higher six-month all-cause readmission and mortality rates. **Conclusions:** ACRS is a frequent and severe complication in AHF. CKD stage and creatinine on admission are key predictors. Early recognition for risk stratification and individualized management are crucial to improving outcomes in this high-risk population.

## 1. Introduction

The term cardiorenal syndrome (CRS) describes the spectrum of interactions between the heart and the kidneys, where acute or chronic dysfunction in one organ triggers acute or chronic dysfunction in the other. Since 2008, the Acute Dialysis Quality Initiative (ADQI) has classified CRS into five subtypes based on disease chronicity and the specific organ involved—a classification still widely used today (Table 1) [1,2].

Acute cardiorenal syndrome (ACRS) is the most common subtype of CRS, characterized by an acute deterioration in cardiac function that subsequently leads to acute kidney injury (AKI). Its incidence ranges from 27% to 50% among all patients with CRS and from 10% to 71% in those hospitalized for acute heart failure (AHF) [1,3].

The pathophysiology of ACRS is primarily driven by hemodynamic changes associated with AHF, including increased central venous and intra-abdominal pressure. The significant rise in these pressures in patients with heart failure (HF) reduces filtration pressure, leading to diminished flow within the kidney capillaries, impaired renal function, and decreased urine output. However, non-hemodynamic mechanisms, such as neurohormonal activation, chronic inflammation, and oxidative stress also contribute to renal dysfunction. Although worsening renal function (WRF) is often attributed to transient hemodynamic shifts, distinguishing between true kidney injury and functional changes remains a challenge, particularly when renal dysfunction is reversible and benign, such as after effective decongestion or the initiation of renin–angiotensin–aldosterone system (RAAS) inhibitors or sodium-glucose cotransporter-2 inhibitors (SGLT2i) [1,4,5].

The diagnosis of ACRS primarily relies on laboratory testing, mainly changes in serum creatinine levels or glomerular filtration rate (GFR). AKI in patients with AHF is traditionally defined as an increase in creatinine of 0.3 mg/dL or more above baseline within 48 h or a 1.5-fold or more increase within 7 days [6]. However, despite its widespread use in renal function assessment, creatinine has inherent limitations, such as the inability to directly evaluate tubular function, delayed rise after AKI, and variability due to factors such as age, sex, ethnicity, and muscle mass [1,7]. Novel biomarkers, such as cystatin C, neutrophil gelatinase-associated lipocalin (NGAL) and kidney injury molecule 1 (KIM-1) have shown promise in recent studies, demonstrating superior prognostic capability for renal injury and patient outcomes compared to creatinine alone. Specifically, NGAL, produced by renal tubular cells during ischemic stress, and KIM-1, expressed on proximal tubular epithelial cells following injury, may enable earlier and more accurate detection of acute kidney injury, facilitating timely clinical interventions. However, further validation in larger patient cohorts is necessary before their widespread clinical implementation can be recommended [1,7,8].

The occurrence of ACRS is associated with increased morbidity and mortality and affected patients are more likely to progress to end-stage CKD or experience further renal function deterioration [9]. Management strategies for ACRS align with those for AHF, emphasizing effective decongestion through diuretics or, in severe cases, renal replacement therapy (RRT) for patients unresponsive to diuretics. Additional therapies include the initiation of angiotensin-converting enzyme inhibitors (ACEi), angiotensin receptor blockers (ARB), beta-blockers, and SGLT2i, all of which have demonstrated benefits in improving outcomes for patients with HF and CKD. In select cases, cardiac assist devices may be utilized to enhance cardiac output [1].

Given the complexities surrounding ACRS, including challenges in diagnosis, prognostication, and optimal therapeutic approaches, our study aimed to identify specific clinical and laboratory factors associated with the occurrence of ACRS. We hypothesized that these factors could serve as predictors of renal dysfunction and clinical deterioration in affected patients. Additionally, we aimed to evaluate the impact of ACRS on renal function deterioration, morbidity, and mortality up to 180 days post-hospitalization, thereby addressing existing knowledge gaps regarding the clinical implications and outcomes associated with ACRS.

## 2. Materials and Methods

### 2.1. Study Population

Patients admitted to the Cardiology Department of Venizelio General Hospital of Heraklion with a diagnosis of acute heart failure were enrolled over a 10-month period, from February to November 2023. A total of 240 patients were initially screened; after applying exclusion criteria, 22 patients were excluded. Reasons for exclusion included dialysis prior to admission (*n* = 3), incomplete laboratory or imaging data (*n* = 16) and early discharge preventing adequate follow-up (e.g., transfer to specialized centers or discharge against medical advice) (*n* = 3). The final study population included 218 patients. 

### 2.2. Inclusion Criteria

All participants met the latest European Society of Cardiology (ESC) criteria for AHF [10]. The diagnosis was based on clinical presentation (e.g., dyspnea, orthopnea, fatigue, lower limb edema, jugular veins distention, and crackles on auscultation) supported by laboratory and imaging findings, including elevated N-terminal pro-B-type natriuretic peptide (NT-proBNP) levels and radiographic evidence of central congestion on chest X-ray.

### 2.3. Exclusion Criteria 

Patients undergoing dialysis before hospital admission, those with incomplete data due to missing laboratory or imaging tests and those discharged early from the Cardiology department, preventing follow-up (e.g., transfer to specialized centers or discharge against medical advice), were excluded, as detailed above.

### 2.4. Methodology

This was a prospective observational study conducted at a single center. Data were collected from patients’ hospital and electronic records after obtaining written informed consent. Most patients were followed up at the Cardiology outpatient clinic after discharge, while the rest were contacted by phone. Their progress was monitored through the electronic system, which records all laboratory results, emergency department (ED) visits, and hospitalizations, including admission and discharge diagnoses from public hospitals across Crete. The definition of ACRS applied in this study was outlined previously in the introduction.

#### 2.4.1. Patient Profile and Laboratory Parameters

Basic demographic characteristics (gender, age), chronic comorbidities (e.g., arterial hypertension, diabetes mellitus, CKD, atrial fibrillation), and the previous New York Heart Association (NYHA) functional class were recorded. CKD was defined as an estimated GFR below 60 mL/min/1.73 m^2^, calculated using the CKD-EPI 2021 equation. Additionally, chronic medication for heart failure (HF), including angiotensin receptor/neprilysin inhibitors (ARNIs), ACEi/ARBs, b-blockers, mineralocorticoid receptor antagonists (MRAs), SGLT2i and furosemide, were documented. Baseline creatinine and corresponding eGFR values were obtained from prior laboratory tests within the previous year to classify the baseline CKD stage (Table A1, Appendix A) [11].

#### 2.4.2. Laboratory Testing

On admission, renal function was assessed through serum creatinine (Cr), serum urea, estimated glomerular filtration rate (eGFR), and serum electrolytes (potassium, sodium). Cardiac biomarkers, including NT-proBNP and high-sensitivity troponin I (hs-cTnI), were also measured. The difference between admission and baseline creatinine (ΔCr1) was recorded.

During hospitalization, hemoglobin, albumin, total protein, glycosylated hemoglobin (HbA1c), and thyroid-stimulating hormone (TSH) were monitored. Renal function was evaluated by tracking serum creatinine and eGFR fluctuations, including their maximum and minimum values. Patients were classified into acute kidney injury (AKI) stages based on the Kidney Disease Improving Outcomes (KDIGO) criteria (Table A2) [11,12,13].

#### 2.4.3. Imaging Parameters

Transthoracic echocardiography was performed within the first 48 h of hospitalization using the Vivid E Series ultrasound system (GE Healthcare, Chicago, IL, USA) by the same specialist. Assessments included left ventricular ejection fraction (LVEF) using the Simpson method, estimation of left ventricular filling pressures (E/E′), tricuspid annular systolic velocity (TV S′), and estimated pulmonary artery systolic pressure (ePASP). Additionally, the presence and severity of valvular heart diseases were assessed through transthoracic echocardiography and recorded systematically.

#### 2.4.4. Endpoints

The primary endpoints were all-cause mortality (in-hospital and at 6 months) and the need for RRT (in-hospital and at 6 months). Secondary endpoints included the number of ED visits, the number of hospital readmissions among survivors, and changes in renal function at 6 months assessed by shifts in KDIGO stage.

#### 2.4.5. Statistical Analysis

Statistical analysis was performed using the IBM SPSS statistical version 29 (IBM Corp., Armonk, NY, USA). Continuous variables were examined for normality of distribution using the Kolmogorov–Smirnov test, along with a review of p-p plots. Quantitative variables with a normal distribution were presented as mean ± standard deviation, while those with a non-normal distribution were presented as median and interquartile range (IQR). For variables with non-normal distribution (e.g., troponin and NT-proBNP values), these were converted to standard values (z-scores). Categorical variables were expressed as frequencies and percentages.

Comparison of quantitative variables between groups were conducted using Student’s *t*-test for normally distributed data or the Mann–Whitney U test for non-normally distributed data. Categorical variables were compared using the chi-square (χ^2^) test, with correlation tables generated. Multivariate logistic regression analysis was performed to identify independent predictors of ACRS occurrence and in-hospital and six-month mortality. Variables included in the multivariate logistic regression were selected based on clinical relevance to ACRS and statistical significance in univariate analysis (*p* < 0.05). Specifically, demographic characteristics (age, sex), comorbidities, renal function parameters, cardiac biomarkers and echocardiographic parameters were entered into the models. Finally, the analysis of covariance (ANCOVA) was employed to examine the effect of selected parameters (e.g., ACRS, CKD stage) on endpoints such as deterioration of renal function at 6 months. The level of statistical significance was defined as a two-sided *p* value less than 0.05 (*p* < 0.05). 

The specifications and objectives of the study followed the ethical guidelines of the Declaration of Helsinki (1975) and are in accordance with the General Data Protection Regulation (GDPR). This study was approved by the Bioethics and Ethics Committee of the Venizelio General Hospital of Heraklion (protocol code 15, 5 August 2022).

## 3. Results

### 3.1. General Characteristics

A total of 218 patients were included in the study, with the majority being male (53.2%) and a median age of 82 years. The most common comorbidities were arterial hypertension (89%), atrial fibrillation (61%), CKD (55.5%) and diabetes mellitus (45%). According to the NYHA functional classification, most patients were in class III (63.8%), and approximately 90% of patients were in CKD stages 2 and 3, as per KDIGO criteria (Table 2). Nearly half of the patients had preserved left ventricular systolic function and were already receiving axis inhibitors and furosemide.

The mean GFR at admission was 57 mL/min/1.73 m^2^, and 51.3% of patients developed ACRS during hospitalization. Table 2 and Table 3 summarize the distribution of comorbidities, NYHA classification, CKD staging, HF medications, and laboratory and imaging parameters at admission. Seventeen percent of patients required inotropes and vasoconstrictors during hospitalization, while two percent required acute RRT. The need for RRT increased to 5.7% within 6 months. In-hospital mortality was 5.5%, rising to 20% at 6 months. Among surviving patients, the average hospital stay was 7 days, with 55.7% visiting the ED at least once and 20% being readmitted for AHF within 6 months (Table 2 and Table 4).

At the 6-month follow-up, re-evaluation of creatinine and eGFR showed a median eGFR of 50 mL/min/1.73 m^2^, indicating a decline of kidney function equal to 1.16 mL/min/1.73 m^2^ per month. A substantial proportion (37.2%) of surviving patients experienced worsening of CKD stage, though most remained in stages 2 and 3 (Table 4).

As shown in Table 4, a statistically significant correlation was observed between CKD stage at admission and after 6 months. Patients in higher CKD stages (>3A) experienced greater deterioration in renal function compared to those in lower stages (<3A).

### 3.2. Patient Characteristics Based on the Occurrence of Acute Cardiorenal Syndrome (ACRS)

As mentioned earlier, 112 out of 218 patients developed ACRS during hospitalization. Statistically significant differences in demographics and comorbidities were observed between patients with and without ACRS, particularly in age, NYHA functional class, and CKD staging. Patients with ACRS were older (82 years vs. 79 years, *p* = 0.014) and had a higher prevalence of coexisting CKD (69.6% vs. 40.6%, *p* < 0.001). ACRS occurred more frequently in patients with a higher NYHA functional class and more advanced CKD stages (Table 2).

Regarding laboratory findings, patients with ACRS exhibited lower baseline eGFR (50 mL/min/1.73 m^2^ vs. 66.5 mL/min/1.73 m^2^, *p* < 0.001) and lower admission eGFR (48.3 mL/min/1.73 m^2^ vs. 66 mL/min/1.73 m^2^, *p* < 0.001). Additionally, they had higher NT-proBNP (7625 pg/mL vs. 5237 pg/mL, *p* = 0.003), higher urea levels (68.5 mg/dL vs. 48 mg/dL, *p* < 0.001), and lower hemoglobin (11.3 g/dL vs. 11.8 g/dL, *p* = 0.019) (Table 3).

Table 5 presents the maximum and minimum creatinine (Cr) and estimated GFR (eGFR) values recorded during hospitalization, along with their corresponding changes, all of which showed statistically significant differences between the two patient groups. However, no significant differences were observed between the groups regarding echocardiographic parameters, HF medications, myocardial injury biomarkers, electrolyte values, or other comorbidities studied (Table 2 and Table 3).

ACRS emerged as a significant risk factor for the studied endpoints, as its occurrence was associated with a substantially higher incidence of mortality both during hospitalization and at 6 months post-discharge. The use of vasoactive agents, including inotropes and vasoconstrictors, was significantly more frequent among patients with ACRS (30.4% vs. 4.7%, *p* < 0.001). Moreover, surviving patients with ACRS exhibited longer hospital stays (8 days vs. 6 days, *p* < 0.001), a higher incidence of renal function deterioration at 6 months (42.5% vs. 24.5%, *p* < 0.001), and a greater frequency of all cause readmissions within 6 months (51.2% vs. 29.8%, *p* = 0.004). However, no significant difference was observed in readmissions specifically related to AHF.

### 3.3. Predictors of Acute Cardiorenal Syndrome

Multivariate analysis, incorporating age, baseline serum creatinine, NT-proBNP, hemoglobin, the difference between baseline and admission serum creatinine (ΔCr1), CKD stage, and NYHA functional class, identified the difference in serum creatinine from baseline (odds ratio [OR] 3.53, 95% confidence interval [CI] 2.02–6.18, *p* < 0.001) and CKD stage (OR 2.30, 95% CI 1.64–3.23, *p* < 0.001) as independent risk factors for the development of ACRS (Table 6).

### 3.4. ACRS and Mortality

A similar approach was used to identify independent risk factors for mortality both during hospitalization and up to 6 months after discharge. The results from univariate and logistic regression analyses of the variables are presented in Table 7 and Table 8, respectively.

Univariate analysis identified several risk factors for in-hospital mortality, including age, the occurrence of ACRS, the stage of AKI, NYHA functional class, admission creatinine and troponin levels, and the need for inotropes (Table 7).

Multivariate analysis revealed that admission troponin levels (OR 2.45, 95% CI 1.17–5.14, *p* = 0.018) and the need for inotropes (OR 10.67, 95% CI 1.52–74.96, *p* = 0.017) were independent predictors of in-hospital mortality.

Several factors were associated with 6-month mortality, including age, the occurrence of ACRS, CKD stage, NYHA functional class, NT-proBNP levels, admission creatinine, albumin, troponin, hemoglobin, peak creatinine during hospitalization, and the need for inotropes. Multivariate analysis revealed that NT-proBNP levels (OR 1.41, 95% CI 1.01–1.99, *p* = 0.048), albumin (OR 0.30, 95% CI 0.11–0.79, *p* = 0.015), troponin I (OR 1.74, 95% CI 1.06–2.66, *p* = 0.028), and the presence of ACRS (OR 2.22, 95% CI 1.01–4.89, *p* = 0.0047) were independent risk factors of mortality at 6 months. Hemoglobin (OR 0.85, 95% CI 0.65–1.00, *p* = 0.05) demonstrated a borderline, yet independent, association with 6-month mortality and was retained in the model due to its known prognostic relevance in this population (Table 8).

### 3.5. Effect of ACRS on Renal Function After 6 Months

Analysis of covariance (ANCOVA) was performed to assess the impact of ACRS occurrence and the baseline CKD stage on the changes in serum creatinine and eGFR levels (ΔGFR) at 6 months post-admission, with the overall need for RRT included as a covariate. ΔGFR was defined as the difference between GFR at 6 months and baseline GFR. 

**Figure 1:**
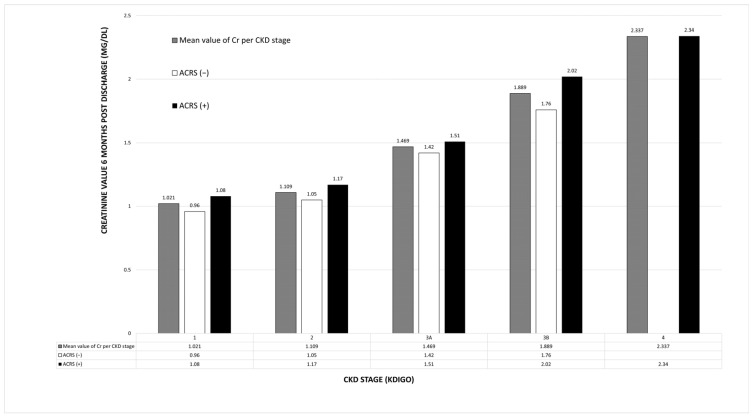
Creatinine values at 6 months based on KDIGO CKD stages and the presence or absence of ACRS. ACRS: Acute cardiorenal syndrome; CKD: Chronic kidney disease; Cr: Creatinine.

The results revealed a statistically significant main effect of KDIGO CKD stage (F = 26.245, *p* < 0.001) and ACRS (F = 3.934, *p* = 0.049) on creatinine levels after 6 months (Figure 1).

**Figure 2:**
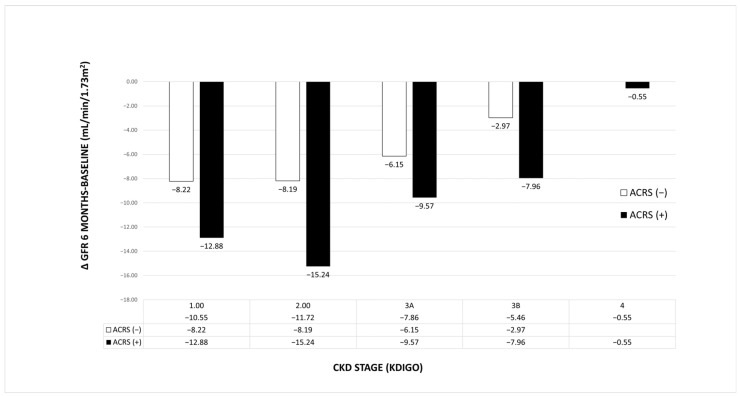
Change in glomerular filtration rate (6 months vs. baseline) according to KDIGO CKD stages and the presence or absence of ACRS. ACRS: Acute cardiorenal syndrome; CKD: Chronic kidney disease; KDIGO: Kidney Disease Improving Global outcomes.

Similarly, for the change in GFR at 6 months, a statistically significant main effect was observed for both KDIGO CKD stage (F = 3.103, *p* = 0.017) and ACRS (F = 6.25, *p* = 0.013, Figure 2). No significant interaction was found between KDIGO CKD stage and ACRS in either analysis. The effect of the covariate, RRT at 6 months, was statistically significant for both creatinine levels at 6 months (*p* < 0.001) and ΔGFR (*p* = 0.001).

The mean creatinine and ΔGFR values at 6 months for each CKD stage and the presence or absence of ACRS, are illustrated in Figure 1 and Figure 2. 

## 4. Discussion

This observational study assessed the risk factors for ACRS and the clinical outcomes of affected patients up to 180 days following admission for AHF. It highlights six main findings: 1. Acute cardiorenal syndrome is a common complication, affecting 51.3% of patients hospitalized for acute heart failure; 2. Advanced CKD stage and greater change in serum creatinine on admission (ΔCr1), both reflecting kidney-driven factors emerged as independent predictors of ACRS; 3. ACRS significantly increases the risk of renal function deterioration and mortality within 6 months post-admission; 4. Acute heart failure even without overt AKI contributes to significant renal function deterioration; 5. In hospital mortality appears primarily heart-driven; and 6. Mortality at 6 months is influenced by multiple factors including cardiac function, anemia and nutritional status.

### 4.1. Incidence of ACRS and Epidemiological Data

Reported incidence rates of ACRS vary widely in the previous literature, ranging from approximately 10% to over 70% among hospitalized AHF patients [1,3]. The relatively high incidence in our cohort (51.3%) is likely explained by the high prevalence of significant comorbidities, including arterial hypertension, diabetes mellitus, CKD and atrial fibrillation—findings consistent with previous studies [14,15]. The observed in-hospital (5.5%) and 6-month mortality rates (20.2%) were comparable to other studies. For example, Lombardi et al. [14] (*n* = 728) reported an in-hospital mortality rate of 8.7%, with a 6-month mortality rate reaching 14%. Likewise, Hu et al. studied 312 patients with AHF and observed an in-hospital mortality rate of approximately 13% [15]. Differences between our findings and these studies may reflect variations in patient populations, disease severity, clinical management strategies and long-term follow-up.

### 4.2. Risk Factors for ACRS

Older age, pre-existing CKD, higher NYHA functional class, and advanced CKD stage emerged as significant risk factors for worsening renal function during hospitalization, as defined by the KDIGO criteria. Patients who developed ACRS had lower baseline and admission GFR values, higher NT-proBNP and urea levels and lower hemoglobin values, underscoring the complex interplay between cardiac and renal function. These findings align with previous literature. For instance, Hu et al., in a study on ACRS risk factors and prognosis in the Chinese population, identified urea, creatinine, eGFR, BNP, hemoglobin, albumin, NYHA classification, and the coexistence of CKD as significant risk factors for ACRS. Among these, reduced eGFR (eGFR < 60 mL/min/1.73 m^2^) and diuretics use were independent risk factors, whereas higher albumin and eGFR levels had a protective effect [15]. Similarly, Ge et al. [16] found that ACRS occurrence was associated with hypertension, coronary artery disease, arterial blood pH, NT-proBNP, serum albumin, and the use of inotropes. However, our study uniquely emphasizes the predictive value of ΔCr1 (the difference between baseline and admission serum creatinine), demonstrating that subtle changes in serum creatinine at admission—an early and readily available clinical marker—significantly influence renal outcomes. Furthermore, these findings support the concept that ACRS occurrence, despite the complex crosstalk between the heart and kidney, is primarily kidney-driven.

### 4.3. Outcomes

The occurrence of ACRS significantly increased the need for hemodynamic support, including inotropes and vasopressors, and was independently associated with higher all-cause mortality and readmission rates at 6 months. Similar associations were demonstrated in prior studies, emphasizing kidney injury as a critical driver of adverse outcomes in AHF [9,15,16]. AKI was linked to increased in-hospital mortality, the need for dialysis [9,15,16], 30-day mortality post-admission [16], prolonged hospital stays [16], and significantly lower rates of renal recovery by discharge [15,16]. Our analysis additionally identified NT-proBNP, albumin, troponin I, and hemoglobin levels as important prognostic factors, reinforcing the necessity of comprehensive clinical management addressing volume status, cardiac function, anemia and nutritional status. This is further supported by many studies demonstrating a direct correlation between these factors and adverse outcomes [17,18,19]. The underlying mechanisms linking ACRS to poor clinical outcomes likely involve persistent neurohormonal activation, sustained inflammation, oxidative stress, and ongoing hemodynamic derangement, collectively exacerbating renal and cardiovascular damage beyond the acute hospitalization phase [1,2,20]. Moreover, distinguishing acute hemodynamically driven changes in glomerular pressure from true renal injury—characterized by structural damage or progression toward chronic tubulointerstitial fibrosis—remains essential for patient management and prognostication [1]. In our study, the occurrence of ACRS accelerated renal function decline and was associated with higher mortality rates, highlighting that ACRS is not always a transient or benign condition. 

### 4.4. ACRS and Renal Function

In this study, renal function was assessed up to 6 months post-hospitalization, revealing that 32.8% of patients experienced worsening renal function, progressing to a higher CKD stage. The occurrence of ACRS had a significant impact, with nearly half (42.5%) of ACRS patients exhibiting a decline in eGFR at 6 months, compared to approximately 25% of those without AKI during hospitalization. 

A notable and novel finding of our study was the significant renal function decline observed at 6 months in patients who initially did not meet ACRS criteria during hospitalization. Nearly 25% of these patients showed substantial renal deterioration, highlighting rapid CKD progression independent of overt AKI. These patients experienced an average eGFR decline of 7 mL/min/1.73 m^2^ over 6 months, significantly exceeding the typical annual decline of 1 mL/min/1.73 m^2^ observed in healthy individuals [4]. This finding highlights the rapid progression of renal dysfunction in patients with heart failure and comorbidities, even without AKI, with ACRS further accelerating this decline. 

The analysis revealed a significant main effect of both CKD stage and ACRS on creatinine levels and changes in eGFR at 6 months, although no significant interaction between these variables was observed in either measurement.

These results underscore the pivotal role of ACRS as a driver of morbidity and mortality in patients hospitalized with AHF. ACRS acts as an independent risk factor for 6-month mortality and has a significant impact on post-discharge renal function. Nearly half of ACRS patients experienced deterioration in CKD stage, along with higher all-cause readmission rates, emphasizing its critical role in patient outcomes.

### 4.5. Limitations

This study has several limitations. First, as a single-center study, its findings may not be fully generalizable to the broader AHF population.

Second, the exclusion of patients with incomplete data introduces potential bias, as these patients may have had different clinical characteristics and outcomes compared to those included in the analysis. This limitation may affect the reliability and generalizability of the study’s findings. Additionally, variability in therapeutic strategies represents another limitation. Despite established guidelines for managing AHF and ACRS, individual physicians’ approaches often vary, potentially influencing clinical outcomes. These differences highlight the impact of overall patient management strategies, which may extend beyond the specific characteristics of the patients themselves.

Another limitation lies in the definition of ACRS, which, in this study, was based on changes in creatinine levels. As noted in the introduction, creatinine is not an ideal marker for fully capturing the scope of AKI, as its fluctuations are often influenced by hemodynamic changes rather than actual tubular damage. Future studies using novel biomarkers (e.g., cystatin C, NGAL, KIM-1) might overcome these limitations [8]. 

In addition, the number of patients requiring renal replacement therapy (RRT) in our cohort was small, limiting the ability to draw meaningful conclusions regarding this outcome. Nonetheless, RRT was included as a predefined endpoint due to its clinical relevance in the context of ACRS.

Furthermore, we did not perform Kaplan–Meier survival analysis, which could have provided additional insight into time-to-event outcomes such as mortality and readmission. This was due to lack of precise timing for all events. We acknowledge this as a methodological limitation and aim to address it in future studies with more complete follow-up data.

Finally, the relatively small sample size limits the robustness of the conclusions and underscores the need for larger, multicenter studies to validate these observations and enhance their statistical power.

## 5. Conclusions

ACRS was a common complication in patients hospitalized with AHF and was primarily kidney-driven, with the difference between admission and baseline creatinine levels and KDIGO-CKD stage as independent predictors. While ACRS significantly contributed to worsening renal function and accelerated kidney decline, renal function deterioration was observed even in patients without ACRS at six months, suggesting a progressive trajectory in this high-risk population. However, ACRS markedly exacerbated this decline, reinforcing its role as a true marker of renal dysfunction rather than a transient hemodynamic shift.

In contrast, in-hospital mortality appeared to be primarily heart-driven, with troponin levels and the need for inotropes/vasopressors emerging as the main independent predictors. Six-month mortality, however, was a multifactorial phenomenon, influenced by anemia, cardiac cachexia, and both cardiac and renal dysfunction. These findings highlight the need for early risk stratification and individualized management strategies to mitigate the adverse consequences of ACRS in AHF patients.

### Future Perspectives

Given the complex interplay between cardiac and renal dysfunction in AHF, future research should prioritize refining risk stratification models to better distinguish true kidney injury from hemodynamic fluctuations. The development of novel biomarkers for early tubular damage, along with individualized therapeutic strategies, may help mitigate the progression of kidney dysfunction and improve long-term outcomes. Additionally, targeted interventions addressing both cardiac and renal drivers of mortality, such as optimized congestion management and nutritional support for cardiac cachexia, could provide a more comprehensive approach to improving survival in this vulnerable population.

## Figures and Tables

**Table 1:** Classification and mechanisms of cardiorenal syndrome [1,2].

Phenotype	Description	Clinical Examples
Type 1–Acute cardiorenal syndrome (ACRS)	Acute heart failure (AHF) resulting in acute kidney injury (AKI)	Acute myocardial infarction, Cardiogenic shock, Acute decompensated heart failure
Type 2–Chronic cardiorenal syndrome	Chronic heart failure resulting in chronic kidney disease (CKD)	Chronic heart failure irrespective of its underlying cause
Type 3–Acute renocardiac syndrome	Acute kidney injury resulting in acute heart failure	Volume overload, Metabolic disturbances in uremia, Inflammatory surge
Type 4–Chronic renocardiac syndrome	Chronic kidney disease resulting in chronic heart failure	Left ventricular hypertrophy, CKD associated cardiomyopathy
Τype 5–Secondary CRS	Systematic process resulting in HF and kidney failure	Amyloidosis, Sepsis, Cirrhosis

ACRS: Acute cardiorenal syndrome; AHF: Acute heart failure; AKI: Acute kidney injury; CKD: Chronic kidney disease, HF: Heart failure.

**Table 2:** Demographic characteristics, comorbidities, chronic medications, and in-hospital outcomes for the entire cohort and based on the occurrence of ACRS.

Parameter	Total *n* = 218	ACRS(+) *n* = 112	ACRS (−) *n* = 106	*p*-Value
Demographics:				
Male sex	116 (53.2%)	62 (55.4%)	54 (50.9%)	0.514
Age > 75 years	161 (73.9%)	90 (80.4%)	71 (67%)	**0.025**
Age (years)	82 (74–86)	82 (78–87)	79 (71–86)	**0.014**
Comorbidities:				
Atrial fibrillation	133 (61%)	67 (59.8%)	66 (62.3%)	0.712
Diabetes mellitus	98 (45%)	54 (48.2%)	44 (41.5%)	0.320
Arterial hypertension	194 (89%)	99 (88.4%)	95 (89.6%)	0.772
Airway disease	68 (31.2%)	36 (32.1%)	32 (30.2%)	0.756
Implantable cardiac defibrillator	9 (4.1%)	5 (4.5%)	4 (3.8%)	0.798
Active smoking	25 (11.5%)	13 (11.6%)	12 (11.3%)	0.947
LVEF: ≤40% 41–49% ≥50%	85 (39%) 27 (12.4%) 106 (48.6%)	48 (42.9%) 10 (8.9%) 54 (48.2%)	37 (34.9%) 17 (16%) 52 (49.1%)	0.229 0.149 0.901
Chronic kidney disease	121 (55.5%)	78 (69.6%)	43 (40.6%)	**<0.001**
CKD stage (KDIGO) 1 2 3A 3Β 4	20 (9.2%) 78 (35.8%) 64 (29.3%) 44 (20.2%) 12 (5.5%)	6 (5.35%) 28 (25%) 35 (31.25%) 32 (28.6%) 11 (9.8%)	14 (13.2%) 50 (47.2%) 29 (27.4%) 12 (11.3%) 1 (0.9%)	**<0.001**
NYHA stage 2 3 4	45 (20.6%) 139 (63.8%) 34 (15.6%)	14 (12.5%) 73 (65.2%) 25 (22.3%)	31 (29.2%) 66 (62.3%) 9 (8.5%)	**<0.001**
**Chronic HF medication:**				
ARNI	9 (5.7%)	3 (2.7%)	6 (5.7%)	0.269
ACEi/ARB	107 (49.1%)	57 (50.9%)	50 (47.2%)	0.583
Β-blocker	133 (61%)	69 (61.6%)	64 (60.4%)	0.852
MRA	51 (23.4%)	29 (25.9%)	22 (20.8%)	0.370
SGLT2i	34 (15.6%)	16 (14.3%)	18 (17%)	0.584
Furosemide	115 (52.8%)	61 (54.5%)	54 (50.9%)	0.603
Furosemide dose (mg)	20 (0–40)	30 (0–55)	20 (0–40)	0.345
**In-hospital complications and mortality up to 6 months:**				
Need for inotropes/vasopressors	39 (17.9%)	34 (30.4%)	5 (4.7%)	**<0.001**
AKI stages (KDIGO) 0 1 2 3	106 (48.6%) 94 (43.1%) 13 (6%) 5 (2.3%)	0 (0%) 94 (83.9%) 13 (11.6%) 5 (4.5%)	106 (100%) 0 (0%) 0 (0%) 0 (0%)	**<0.001**
Total hospital days (*n* = 206)	7 (5–10)	8 (6–12)	6 (5–8)	**<0.001**
Need for RRT	4 (1.8%)	4 (3.6%)	0 (0%)	**0.05**
Death (all-cause)	12 (5.5%)	10 (8.9%)	2 (1.9%)	**0.023**
Cardiovascular death	8 (3.7%)	6 (5.4%)	2 (1.9%)	0.173
6-month mortality	44 (20.2%)	32 (28.6%)	12 (11.3%)	**0.002**

ACEi: Angiotensin-converting enzyme inhibitors; ACRS: Acute cardiorenal syndrome; AKI: Acute kidney injury; ARB: Angiotensin receptor blocker; CKD: Chronic kidney disease; KDIGO: Kidney Disease Improving Global outcomes; LVEF: Left ventricular ejection fraction; MRA: Mineralocorticoid receptor antagonist; NYHA: New York Heart Association; RRT: Renal replacement therapy; SGLT2i: Sodium-glucose cotransporter-2 inhibitors; ARNI: Angiotensin receptor/neprilysin inhibitor.

**Table 3:** Laboratory tests and echocardiographic parameters for the entire cohort, as well as based on the occurrence of ACRS.

Parameter	Total (*n* = 218)	ACRS (+) (*n* = 112)	ACRS (−) (*n* = 106)	*p*-Value
Laboratory evaluation on admission:				
GFR (mL/min/1.73 m^2^)	57 ± 21.5	48.3 ± 20	66 ± 19	**<0.001**
Cr (mg/dL)	1.14 (0.94–1.49)	1.36 (0.86–1.78)	1.02 (0.86–1.22)	**<0.001**
Baseline Cr (mg/dL)	1.1 (0.9–1.4)	1.2 (1–1.6)	1 (0.9–1.22)	**<0.001**
Baseline GFR (mL/min/1.73 m^2^)	56 (44–78)	50 (39–64.75)	66.5 (54–80)	**<0.001**
Urea (mg/dL)	56.5 (44–85)	68.5 (50 -103.5)	48 (40–63)	**<0.001**
NT-proBNP (pg/mL)	5972 (3126–11,345)	7625 (3662–14,898)	5237 (2797–8974)	**0.003**
Hs-cTnΙ (pg/mL)	26.6 (13.13–68)	31.29 (14.77–94.73)	24.7 (12.53–51.67)	0.164
Sodium (mEq/L)	139 (137–141)	139 (136–141)	139 (138–141)	0.057
Potassium (mEq/L)	4.53 ± 0.59	4.56 ± 0.61	4.5 ± 0.58	0.476
In hospital laboratory evaluation:				
HbA1c (%)	6 (5.6–6.8)	6.1 (5.6–6.9)	5.9 (5.6–6.62)	0.246
Hb (g/dL)	11.6 ± 1.8	11.3 ± 1.8	11.8 ± 1.8	**0.019**
Albumin (g/dL)	4 (3.8–4.2)	4 (3.8–4.3)	4 (3.8–4.2)	0.949
Total protein (g/dL)	6.7 (6.4–7.2)	6.7 (6.3–7.2)	6.8 (6.4–7.2)	0.770
TSH (mU/L)	1.24 (0.72–2.16)	1.42 (0.72–2.22)	1.17 (0.72–2.09)	0.567
**Echocardiographic parameters:**				
LVEF (%)	45 (30–55)	45 (25–55)	45 (30–55)	0.276
ePASP (mmHg)	40 (35–50)	40 (35–50)	40 (35–55)	0.437
E/E′	15 (12–20)	15 (12–20)	14.5 (12–20.5)	0.846
TV S′ (cm/s)	10.5 (9–12)	11 (9–12)	10.5 (9–12)	0.807
**Tricuspid regurgitation:** No/mild Moderate Severe	128 (58.7%) 52 (23.9%) 38 (17.4%)	69 (61.6%) 27 (24.1%) 16 (14.3%)	59 (55.7%) 25 (23.6%) 22 (20.8%)	0.440
**Mitral regurgitation:** No/mild Moderate Severe	136 (62.4%) 66 (30.3%) 16 (7.3%)	70 (62.5%) 36 (32.1%) 6 (5.4%)	66 (62.3%) 30 (28.3%) 10 (9.4%)	0.473
**Mitral stenosis** No/mild Moderate Severe	211 (96.8%) 4 (1.8%) 3 (1.4%)	108 (96.4%) 3 (2.7%) 1 (0.9%)	103 (97.2%) 1 (0.9%) 2 (1.9%)	0.512
**Aortic regurgitation:** No/mild Moderate Severe	195 (89.4%) 21 (9.7%) 2 (0.9%)	99 (88.4%) 12 (10.7%) 1 (0.9%)	96 (90.6%) 9 (8.5%) 1 (0.9%)	0.846
**Severe aortic stenosis**	28 (12.8%)	13 (11.6%)	15 (14.2%)	0.575

ACRS: Acute cardiorenal syndrome; Cr: Creatinine; ePASP: Estimated pulmonary artery systolic pressure; GFR: Glomerular filtration rate; Hb: Hemoglobin; HbA1c: Glycosylated hemoglobin; Hs-cTnI: High-sensitive cardiac troponin-I; LVEF: Left ventricular ejection fraction; NT-proBNP: N-terminal pro-B-type natriuretic peptide; TSH: Thyroid-stimulating hormone; TV S′: Lateral tricuspid annular systolic velocity, E/E′: Estimation of left ventricular filling pressures.

**Table 4:** Outcomes in 6 months for surviving patients.

Outcomes for Survivors at 6 Months				
Parameter:	Total (*n* = 174)	ACRS (+) (*n* = 80)	ACRS (−) (*n* = 94)	*p*-Value
6 months				
Emergency department visit	97 (55.7%)	50 (62.5%)	47 (50%)	0.098
Readmission (all-cause)	69 (39.7%)	41 (51.2%)	28 (29.8%)	**0.004**
Number of readmissions (all-cause)	0 (0-1)	0.5 (0–2)	0 (0–1)	**0.005**
Readmission due to AHF	35 (20.1%)	17 (21.3%)	18 (19.1%)	0.730
CKD stage (KDIGO) 1 2 3A 3B 4 5	12 (6.9%) 49 (28.2%) 43 (24.7%) 49 (28.2%) 16 (9.2%) 5 (2.8%)	3 (3.75%) 13 (16.25%) 15 (18.75%) 30 (37.5%) 14 (17.5%) 5 (6.25%)	9 (9.6%) 36 (38.3%) 28 (29.8%) 19 (20.2%) 2 (2.1%) 0 (0%)	**<0.001**
Renal function deterioration (CKD stage)	57 (32.8%)	34 (42.5%)	23 (24.5%)	**0.012**
New baseline Cr (mg/dL)	1.39 ± 0.4	1.64 ± 0.77	1.19 ± 0.39	**<0.001**
New baseline GFR (mL/min/1.73 m^2^)	50 (36–66)	41 (30–56.75)	59 (45–74)	**<0.001**
ΔGFR (6M–BASELINE)	−8 ± 11	−10 ± 13	−7 ± 9	**0.03**
RRT	10 (5.7%)	7 (8.8%)	3 (3.2%)	0.116

AHF: Acute heart failure; CKD: Chronic kidney disease; Cr: Creatinine; GFR: Glomerular filtration rate; KDIGO: Kidney Disease Improving Global outcomes; RRT: Renal replacement therapy; ΔGFR (−BASELINE): difference between glomerular filtration rate 6 months post hospitalization and initial baseline glomerular filtration rate value.

**Table 5:** Creatinine and estimated glomerular filtration rate values based on the occurrence of in-hospital ACRS.

In-Hospital Renal Function:				
Parameter:	Total (*n* = 218)	ACRS (+) (*n* = 112)	ACRS (−) (*n* = 106)	*p*-Value
Maximum Cr (mg/dL)	1.5 (1.15–2)	1.92 (1.58–2.53)	1.17 (1–1.4)	**<0.001**
Minimum eGFR (mL/min/1.73 m^2^)	42 (28–57.25)	29.5 (23–38.75)	55 (46–65.25)	**<0.001**
Minimum Cr (mg/dL)	1.05 (0.87–1.35)	1.18 (0.93–1.55)	0.96 (0.8–1.14)	**<0.001**
Maximum eGFR (mL/min/1.73 m^2^)	63.5 ± 22	56 ± 21.8	71 ± 19.5	**<0.001**
ΔCr (mg/dL)	0.37 (0.21–0.66)	0.64 (0.44–0.98)	0.21 (0.14–0.29)	**<0.001**
ΔGFR (mL/min/1.73 m^2^)	17 (10–26)	22 (13–33)	12.5 (7–20)	**<0.001**
ΔCr1 [admission-baseline] (mg/dL)	0.03 (−0.042–0.18)	0.09 (−0.02–0.315)	−0.01 (−0.08–0.08)	**<0.001**
Admission ΔCr [Admission Cr − Min Cr ≥ 0.3 mg/dL]	37 (17%)	31 (27.7%)	6 (5.7%)	**<0.001**

Admission ΔCr: Difference between admission creatinine value and the minimum creatinine value recorded during hospitalization; Cr: Creatinine; GFR: Glomerular filtration rate; ΔCr: difference between maximum creatinine and minimum creatinine value during hospitalization; ΔCr1: difference between admission creatinine value and baseline creatinine value; ΔGFR: difference between maximum glomerular filtration rate and minimum glomerular filtration rate value during hospitalization; eGFR: Estimated glomerular filtration rate.

**Table 6:** Predictors of acute cardiorenal syndrome occurrence.

	Univariate			Multivariate		
Parameter	OR	95% CI	*p*	OR	95% CI	*p*
Age (1 year)	1.04	1.01–1.07	0.020	-	-	-
CKD stage (1 stage)	2.10	1.56–2.83	0.000	2.30	1.64–3.23	0.000
ΝYHA class (1 class)	2.47	1.52–4.03	0.000			
ΔCr1 [ADMISSION − BASΕLINE] (1 SD)	2.898	1.81–4.63	0.000	3.53	2.02–6.18	0.000
ΝΤ-proBNP (pg/mL) (1 SD)	1.71	1.23–2.37	0.001			
Admission Cr (1 mg/dL)	11.37	4.72–23.84	0.000			
Hb (1 g/dL)	0.84	0.72–0.97	0.020	-	-	-

95% CI: 95% Confidence interval; CKD: Chronic kidney disease; Cr: Creatinine; Hb: Hemoglobin; NT-proBNP: N-terminal pro-B-type natriuretic peptide; NYHA: New York Heart Association; ΔCr1: Difference between admission creatinine value and baseline (before admission) creatinine value.

**Table 7:** Predictors of in-hospital mortality.

	Univariate			Multivariate		
Parameter	OR	95% CI	*p*	OR	95% CI	*p*
Age (years)	1.12	1.02–1.23	0.017	-	-	-
ACRS (yes = 1, no = 0)	5.10	1.09–23.84	0.038	-	-	-
ΝYHA (per 1 class)	3.20	1.16–48.87	0.025	-	-	-
AKI (per 1 stage)	3.14	1.58–6.26	0.001	-	-	-
Inotropes/Vasopressors (yes = 1, no = 0)	30.52	6.36–146.6	0.000	10.67	1.52–74.96	0.017
Troponin I (pg/mL) (1 SD)	2.75	1.67–4.52	0.000	2.452	1.17–5.14	0.018

95% CI: 95% Confidence interval; ACRS: Acute cardiorenal syndrome; Admission ΔCr: difference between admission creatinine value and the minimum creatinine value recorded during hospitalization; AKI: Acute kidney injury; NYHA: New York Heart Association; OR: Odds ratio; SD: Standard deviation.

**Table 8:** Predictors of 6-month mortality post hospitalization.

	Univariate			Multivariate		
Parameter	OR	95% CI	*p*	OR	95% CI	*p*
Age (1 year)	1.05	1.01–1.10	0.015	-	-	-
ACRS (yes = 1, no = 0)	3.13	1.51–6.48	0.002	2.22	1.01–4.89	0.0047
CKD stage (KDIGO) (1 stage)	2.10	1.56–2.83	0.000	-	-	
AKI stage (1 stage)	1.90	1.21–2.99	0.000			
ΝYHA (1 stage)	2.53	1.40–4.55	0.002	-	-	-
NT-proBNP (pg/mL) (1 SD)	1.75	1.30–2.37	0.000	1.41	1.01–1.99	0.048
Admission Cr (1 mg/dL)	11.37	4.72–23.84	0.000			
Albumin (1g/dL)	0.29	0.12–0.70	0.006	0.30	0.11–0.79	0.015
Maximum Cr (1 mg/dL)	1.94	1.25–3.01	0.003	-	-	-
Troponin-I (pg/mL) (1 SD)	1.77	1.15–2.72	0.009	1.74	1.06–2.66	0.028
Hb (1 g/dL)	0.75	0.62–0.92	0.006	0.85	0.65–1	0.050
Inotropes/Vasopressors (yes = 1, no = 0)	3.75	1.76–7.98	0.001			

95% CI: 95% Confidence Interval; ACRS: Acute cardiorenal syndrome; AKI: Acute kidney injury; CKD: chronic kidney disease; Cr: Creatinine; Hb: Hemoglobin; KDIGO: Kidney Disease Improving Global outcomes; NT-proBNP: N-terminal pro-B-type natriuretic peptide; NYHA: New York Heart association; OR: Odds ratio; SD: Standard deviation.

## Data Availability

The data supporting the findings of this study are not publicly available due to ethical considerations and patient confidentiality. Access to the dataset may be provided upon reasonable request to the corresponding author, subject to approval by the Bioethics and Ethics Committee of the Venizelio General Hospital of Heraklion.

## Abbreviations

The following abbreviations are used in this manuscript:

95% CI	95% Confidence interval
ACEi	Angiotensin-converting enzyme inhibitor
ACRS	Acute cardiorenal syndrome
Admission ΔCr	Difference between admission creatinine value and the minimum creatinine value recorded during hospitalization
ADQI	Acute Dialysis Quality Initiative
AHF	Acute heart failure
AKI	Acute kidney injury
ANCOVA	Analysis of covariance
ARBs	Angiotensin receptor blocker
ARNIs	Angiotensin receptor/neprilysin inhibitor
CKD	Chronic kidney disease
CRS	Cardiorenal syndrome
ED	Emergency department
eGFR	Estimated Glomerular filtration rate
GFR	Glomerular filtration rate
ESC	European Society of Cardiology
GDPR	General Data Protection Regulation
Hb	Hemoglobin
HbA1c	Glycosylated hemoglobin
HF	Heart Failure
Hs-cTnI	High-sensitive troponin-I
IQR	Interquartile range
KIM-1	Kidney injury molecule 1
KDIGO	Kidney Disease Improving Global Outcomes
LVEF	Left ventricular ejection fraction
MRAs	Mineralocorticoid receptor blockers
NGAL	Neutrophil gelatinase-associated lipocalin
NT-proBNP	N-terminal pro-B-type natriuretic peptide
NYHA	New York Heart Association
OR	Odds Ratio
RAAS	Renin–Angiotensin–Aldosterone System
RRT	Renal replacement therapy
SD	Standard deviation
SGLT2i	Sodium-glucose cotransporter-2 inhibitors
TSH	Thyroid-stimulating hormone
TV S′	Lateral tricuspid annular systolic velocity
WRF	Worsening renal function
ΔCr	Difference between maximum creatinine and minimum creatinine value during hospitalization
ΔCr1	Difference between admission creatinine value and baseline creatinine value
ΔGFR	Difference between maximum glomerular filtration rate and minimum glomerular filtration rate value during hospitalization
ΔGFR (6M-BASELINE)	Difference between glomerular filtration rate 6 months post hospitalization and baseline glomerular filtration rate value

## Appendix A

### Appendix A.1. Definition and Classification of Acute and Chronic Kidney Disease

Chronic kidney disease:

Chronic kidney disease (CKD) is defined as the presence of structural or functional kidney abnormalities persisting for at least three months. CKD is classified based on three key parameters: the underlying cause, the glomerular filtration rate (GFR; stages G1–G5), and the presence or absence of albuminuria (stages A1–A3). These components play a critical role in determining the severity of the disease and assessing the risk profile of affected patients. The classification system based on GFR is outlined in Table A1 [3].

**Table A1:** Stages of CKD according to KDIGO criteria [3].

GFR Categories	GFR (mL/min/1.73 m^2^)	Description
**G1**	>90	Normal or high
**G2**	60–89	Mildly decreased
**G3a**	45–59	Mildly to moderately decreased
**G3b**	30–44	Moderately to severely decreased
**G4**	15–29	Severy decreased
**G5**	<15	Kidney failure

Acute kidney injury:

The term acute kidney injury (AKI) refers to the sudden onset of functional or structural damage to the kidneys, occurring over a period of hours to a few days. Several diagnostic criteria—RIFLE, AKIN, and KDIGO—have been established to define AKI based on changes in serum creatinine levels, estimated glomerular filtration rate (eGFR), and urine output [1]. According to the 2012 Kidney Disease: Improving Global Outcomes (KDIGO) guidelines, AKI is defined by the presence of at least one of the following:

- An increase in serum creatinine of 0.3 mg/dL or more within 48 h, or
- An increase in serum creatinine to more than 1.5 times the baseline value, known or
- Presumed to have occurred within the preceding seven days, or
- Urine output of less than 0.5 mL/kg/hour for a duration of six hours
- Accordingly, the classification of AKI, as outlined in the 2012 KDIGO guidelines, is presented in Table A2 [1,2].

**Table A2:** AKI staging based on the KDIGO criteria.

AKI Stage	Serum Creatinine Criteria	Urine Output Criteria
**1**	Serum Cr increase ≥ 0.3 mg/dL within 48 h or Serum Cr increase ≥ 1.5–2 fold from baseline	<0.5 mL/kg/h for 6 consecutive hours
**2**	Serum Cr increase ≥ 2–3 fold from baseline	<0.5 mL/kg/h for 12 h
**3**	Serum Cr increase ≥ 3 fold from baseline or Serum creatinine increase ≥ 4 mg/dL or Initiated on RRT (irrespective of stage at time of initiation)	<0.3 mL/kg/h for 24 h or anuria for 12 h
